# Pediatric Pulmonary Function Testing in COVID-19 Pandemic and Beyond. A Position Statement From the Hellenic Pediatric Respiratory Society

**DOI:** 10.3389/fped.2021.673322

**Published:** 2021-05-21

**Authors:** Sotirios Fouzas, Dimos Gidaris, Nikolaos Karantaglis, Harry Opsimos, Emmanouil I. Alexopoulos, Konstantinos Douros, Fotios Kirvassilis, Emmanouil Paraskakis, Michael B. Anthracopoulos, Markos Marangos, Ioannis Tsanakas, the Hellenic Pediatric Respiratory Society

**Affiliations:** ^1^Pediatric Respiratory Unit, University Hospital of Patras, Patras, Greece; ^2^School of Medicine, University of Nicosia, Nicosia, Cyprus; ^3^Pediatric Pulmonology Unit, 3rd Department of Pediatrics, Hippokration Hospital of Thessaloniki, Thessaloniki, Greece; ^4^Private Pediatric Pulmonologist, Athens, Greece; ^5^Pediatric Respiratory Division, University Hospital of Larissa, Larissa, Greece; ^6^Pediatric Respiratory and Allergy Unit, 3rd Department of Pediatrics, Attikon Hospital, Athens, Greece; ^7^Pediatric Respiratory Unit, University Hospital of Heraklion, Iraklio, Greece; ^8^Department of Infectious Diseases, University Hospital of Patras, Patras, Greece

**Keywords:** COVID-19, spirometry, lung function testing, children, recommendation

## Abstract

As the COVID-19 pandemic is still evolving, guidelines on pulmonary function testing that may dynamically adapt to sudden epidemiologic changes are required. This paper presents the recommendations of the Hellenic Pediatric Respiratory Society (HPRS) on pulmonary function testing in children and adolescents during the COVID-19 era. Following an extensive review of the relevant literature, we recommend that pulmonary function tests should be carried out after careful evaluation of the epidemiologic load, structured clinical screening of all candidates, and application of special protective measures to minimize the risk of viral cross infection. These principles have been integrated into a dynamic action plan that may readily adapt to the phase of the pandemic.

## Introduction

Pulmonary function testing is instrumental for the diagnosis and follow up of pediatric chronic respiratory disorders, including asthma, cystic fibrosis (CF), primary ciliary dyskinesia, and restrictive and interstitial lung diseases (1, 2). Pulmonary function tests (PFTs) are also essential for the evaluation of lung involvement in systemic disorders, preoperative risk assessment and enrolment on transplant lists (1).

Bacterial or viral cross infection is possible to occur in the pulmonary function laboratory (3). Although the European Respiratory Society (ERS) and the American Thoracic Society (ATS) have issued special guidelines on infection control during pulmonary function testing, the current coronavirus disease 2019 (COVID-19) pandemic has raised additional concerns: the load of severe acute respiratory syndrome coronavirus 2 (SARS-CoV-2) in the respiratory secretions of infected individuals is high (4), therefore, breathing maneuvers that lead to aerosol formation (e.g., cough, sneezing, or forced expiration) may favor its spread and increase the risk of cross contamination (5).

During the initial phase of COVID-19 pandemic, national and international health authorities advised against routine pulmonary function testing (6). However, as many countries entered the post-peak phase of the pandemic and the restriction measures were gradually easing in the communities, the need to restart specialized health services resurfaced (6, 7). Thus, most national and international scientific bodies amended their initial recommendations and have reintroduced PFTs in routine clinical practice.

Yet, the COVID-19 pandemic is still evolving, and its epidemiological features remain labile and unpredictable (8, 9). More stringent mitigation measures may again be required in the future to control the spread of the disease (9) and such measures will undoubtedly affect the operation of pulmonary function laboratories. Moreover, lung function anomalies and psychological sequelae are not uncommon in individuals recovering from COVID-19 (10, 11) which implies that a constantly increasing number of patients will require close lung function monitoring during the pandemic (12). Therefore, guidelines on pulmonary function testing that include specific action plans and may dynamically adapt to sudden epidemiologic changes, are required.

In this paper we briefly review the current national and international guidelines and we present the recommendations of the Hellenic Pediatric Respiratory Society (HPRS) on pulmonary function testing in children and adolescents during the COVID-19 era and beyond. This position statement is based on the consensus of the authors, who are members of the HPRS and may be subject to changes as the pandemic evolves.

## Review of the Literature

To date, there are no reports in the literature (PubMed, Medline Plus, Embase) confirming SARS-CoV-2 transmission in the pulmonary function laboratory. However, PFTs that involve forced expiratory maneuvers (e.g., spirometry) or respiratory maneuvers in a restricted space (e.g., body plethysmography) or expiration of high minute volumes (e.g., cardiopulmonary exercise testing–CPET), may favor bacterial and viral spread (3) (Table 1). SARS-CoV-2 cannot be an exception; on the contrary, its particularly high load in respiratory secretions (4) favors the transmission of the virus through aerosols (5). Hence, most authorities have issued special guidelines for the management of COVID-19 patients with respiratory symptoms, including the recommendation to avoid the use of nebulizers (13, 14).

**Table 1:** Pulmonary function tests and risk of cross infection.

**Test**	**Risk**
Static lung volumes & capacities Tidal Breathing measurements	Theoretically low (no data available) No forced breathing maneuvers & use of bacterial filter
Forced spirometry	Moderate to high Forced expiration generates aerosols Although bacterial filter is used, droplets may escape around the mouthpiece or due to cough
Fractional exhaled nitric oxide (FeNO) measurement	Theoretically low (no data available) No forced breathing maneuvers & use of bacterial filter Potential circuit contamination
Forced oscillation	Theoretically low (no data available) No forced breathing maneuvers & use of bacterial filter Potential circuit contamination
Multiple breath washout Measurement of lung volumes with helium dilution technique Measurement of lung diffusion capacity	Theoretically low (no data available) No forced breathing maneuvers & use of bacterial filter Potential circuit contamination
Body plethysmography	Moderate to high Respiratory maneuvers (± forced) in closed space Potential circuit and “body-box” contamination
Bronchoprovocation tests (methacholine, mannitol)	High Provocation of cough and generation of aerosols
Bronchodilator reversibility testing	High if nebulizers are used Low with pMDIs and use of holding chamber Potential holding chamber contamination
Cardiopulmonary exercise testing	High (no data available) Exhalation of large volumes of air per minute without a bacterial filter

During the first phases of the pandemic, PFTs were universally suspended to control the spread of the disease (5). However, as the pandemic unfolded, ERS (15), ATS (16) and several national respiratory societies (17–32) have issued detailed recommendations (Table 2), of which the main points could be summarized as follows:

- The value of pulmonary function testing is reiterated together with the need to prevent infection spread to patients and staff (17–32).
- It is universally recommended to avoid pulmonary function testing in patients infected with COVID-19 (17–32).
- It is not generally recommended to routinely test for COVID-19 prior to perform PFTs (17–23, 27–32).
- It is strongly advised to perform structured clinical screening, including measurement of body temperature and assessment of relevant history and symptoms, to all candidates for pulmonary function testing (17–32).
  - In case of positive clinical screening, it is recommended to postpone pulmonary function testing for at least 14 days (15).
  - In case of negative clinical screening, it is recommended to carefully select the patients who will undergo PFTs. The selection criteria may be:
  * *Stringent*: PFTs are performed only when necessary, for e.g., in patients for whom urgent management decisions must be taken (22– 25, 29, 31, 32).
  * *Less stringent*: PFTs may be performed in patients with CF, poorly controlled asthma, initial asthma diagnosis, asthma that requires treatment modification, patients with chronic lung diseases that require therapeutic interventions, patients with interstitial or restrictive lung disorders and for pre-operative evaluation (15–21, 26–28, 30).
- Some experts (15, 16, 28) suggest that the phase of the pandemic should be taken into account: PFTs are suspended or performed with stringent selection criteria when the epidemiologic load is high (i.e., around the peak phase of the pandemic), while more loose criteria should be adopted when the epidemiologic load is low or declines.
- Spirometry is the only test that can be performed even when the epidemiologic load is moderate, provided that stringent selection criteria are applied (17–32).
- In any phase of the pandemic, PFTs are carried out with special protective measures for all involved parties (laboratory staff, patients, accompanying family members) (17–32).

**Table 2:** National and international recommendations on performing PFTs during the COVID-19 pandemic.

**Society**	**Date**	**Recommendation**	**Prerequisites**			**Comments**
			**Negative test**	**Clinical screening**	**Patient selection criteria**	
European respiratory society (15)	3/2020	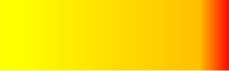	No	Yes	Less stringent	Recommendation by expert group. The guideline is adjusted according to the phase of the pandemic. Full text
American thoracic society (16)	3/2020	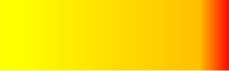	No	Yes	Less stringent	No adjustment according to the phase of the pandemic. Short statement
British thoracic society (17)	4/2020	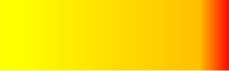	No	–	Less stringent	Recommendation by expert group. The guideline is adjusted according to the phase of the pandemic (up to its peak). Full text
Thoracic society of Australia and New Zealand (18)	5/2020	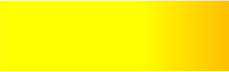	No	Yes	Less stringent	No adjustment according to the phase of the pandemic (refers only to post-peak phase). Short statement
Italian respiratory society (19)	5/2020	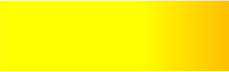	No	Yes	Less stringent	Recommendation by expert group. No adjustment according to the phase of the pandemic (refers only to post-peak phase). Full text
Spanish scientific societies (20)	3/2020	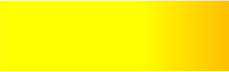	No	Yes	Less stringent	Recommendation by expert group. No adjustment according to the phase of the pandemic. Full text
French respiratory society (21)	3/2020	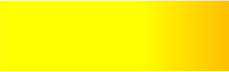	No	Yes	Less stringent	Recommendation by expert group. No adjustment according to the phase of the pandemic. Full text
Irish thoracic society (22)	3/2020	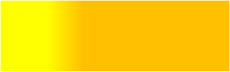	No	Yes	Stringent	No adjustment according to the phase of the pandemic. Short statement
Respiratory branch of the Chinese medical association (23)	3/2020	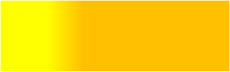	No	Yes	Stringent	Recommendation by expert group. No adjustment according to the phase of the pandemic. Full text
Canadian thoracic society (24)	7/2020	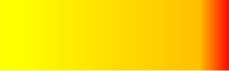	Yes	Yes	Stringent	Recommendation by expert group. No adjustment according to the phase of the pandemic (refers only to post-peak phase). Full text
Russian respiratory society (25)	5/2020	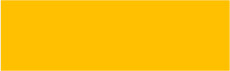	No	Yes	Stringent	Recommendation by expert group. No adjustment according to the phase of the pandemic. Full text
Swiss society for pulmonology (26)	11/2020	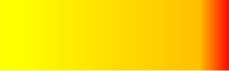	No	–	Less stringent	Recommendation by expert group. No adjustment according to the phase of the pandemic. Short statement
Latin-American society of respiratory physiology (27)	6/2020	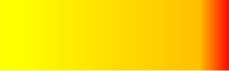	No	Yes	Less stringent	Recommendation by expert group. No adjustment according to the phase of the pandemic. Full text
Argentinian association of respiratory medicine (28)	1/2021	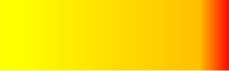	No	Yes	Less stringent	Recommendation by expert group. The guideline is adjusted according to the epidemiological load. Full text
Turkish thoracic society (29)	5/2020	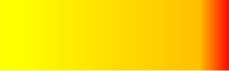	No	Yes	Stringent	Recommendation by expert group. No adjustment according to the phase of the pandemic. Full text
**Pediatric guidelines**						
ATS/ERS (30)	11/2020	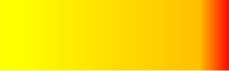	Yes	Yes	Less stringent	Recommendation by expert group. No adjustment according to the phase of the pandemic. Full text
India (experts consortium) (31)	11/2020	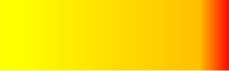	No	Yes	Stringent	Recommendation by expert group. Part of a general pediatric respiratory recommendation. No adjustment according to the phase of the pandemic
Italian pediatric respiratory society (32)	4/2020	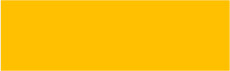	No	Yes	Stringent	Recommendation by expert group. No adjustment according to the phase of the pandemic. Full text

*
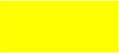
: Most PFTs with caution 
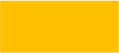
: Only spirometry ± gas dilution techniques 
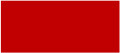
: Suspension of all PFTs*.

*PFTs, pulmonary function tests; CF, cystic fibrosis*.

Most of these recommendations are expert group consensuses (15, 17, 19–21, 23–32). To date, pediatric-specific guidelines have been issued by the ERS/ATS (30), a consortium of pediatric pulmonology experts from India (31), and the Italian Pediatric Respiratory Society (32). The latter suggests that during the COVID-19 pandemic pulmonary function testing should be limited to spirometry in patients selected with stringent criteria. However, it should be mentioned that this recommendation was based on epidemiologic data collected up to March 2020 in Italy; more recently, the Italian Respiratory Society recommended the reintroduction of all PFTs in clinical practice with less stringent patient selection criteria (19). The Indian guideline also adopts stringent patient selection criteria and recommends reduction of PFTs to the absolutely necessary (31). Converselly, an ATS/ERS webinar on the international perspectives on resuming PFTs during COVID-19 (32), concluded that laboratories may reopen in areas with decreased number of new COVID-19 cases, but with reduced activity at the beginning; no specific patient selection criteria or action plans related to the epidemiological load were included (32).

## HPRS Recommendation

Based on the existing evidence, we recommend that the decision to perform PFTs during the COVID-19 pandemic should be based on the following four principles:

- PFTs postponement in children and adolescents with active SARS-CoV-2 infection.
- Evaluation of the epidemiologic load (phase of the pandemic) to decide whether and which PFTs can be performed in non-infected individuals.
- Structured clinical screening of all eligible children prior to performing PFTs.
- Application of special protective measures to minimize the risk of SARS-CoV-2 cross infection.

**Figure 1:**
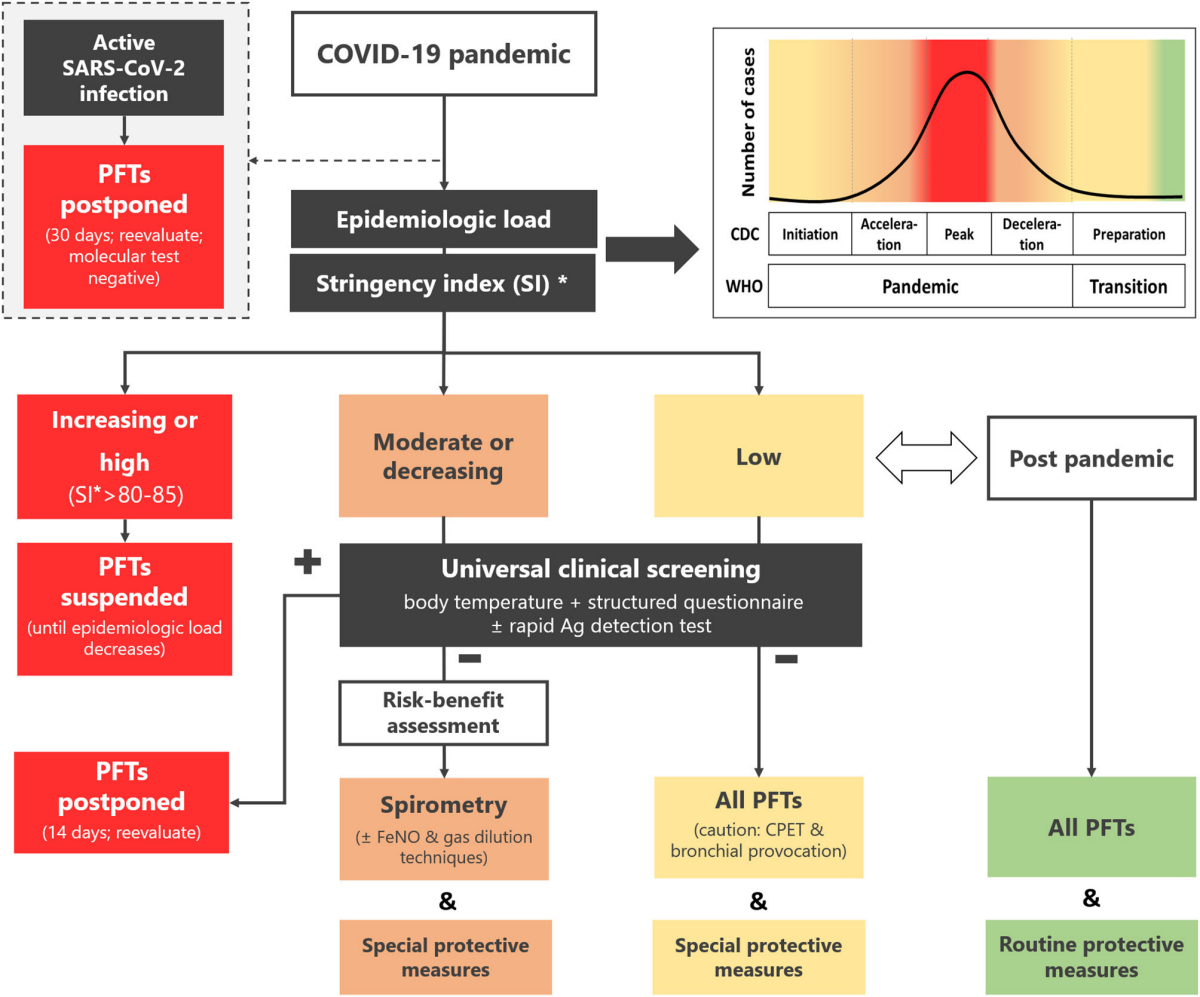
Recommendation of the Hellenic Pediatric Respiratory Society regarding pulmonary function testing in children and adolescents during the COVID-19 pandemic and beyond. *Available online at: https://ourworldindata.org/covid-government-stringency-index#containment-and-health-index. PFTs, pulmonary function tests; CDC, Centers for Disease Control and Prevention; WHO, World Health Organization; FeNO, fraction of expired NO; CPET, cardiopulmonary exercise testing; SI, stringency index.

An outline of the HPRS recommendation is presented in Figure 1.

Specifically:

### PFTs Postponement in Children and Adolescents With SARS-CoV-2 Infection

In children and adolescents with proven SARS-CoV-2 infection (positive molecular test) all PFTs should be postponed for at least 30 days. Following this period, PFTs may be carried out if deemed necessary, provided that the patient is asymptomatic and at least one SARS-CoV-2 molecular test is negative (33).

### Evaluation of the Epidemiologic Load

The epidemiologic load of SARS-CoV-2 depends on the phase of the pandemic on national, regional, or even local level (Figure 1). Since these data are under constant review by the regional monitoring bodies and national health authorities, the laboratory staff should actively seek daily updates and guidance from local coordinators. Moreover, online tools that track and compare policy responses around the world (34) can be used as a quick reference regarding the actual epidemiological load of the country/region.

The following scenarios may be distinguished:

- *Increasing or high epidemiologic load*.

Corresponds to the period from the initiation of the *Pandemic* phase up to after its peak (WHO) or to *Initiation, Acceleration* and *Peak* stages of the pandemic (CDC) (Figure 1). Extended restriction measures (high stringency index, e.g., >85) (33) are imposed by the authorities.

Recommendation: All PFTs are suspended until the epidemiologic load decreases.

- *Moderate or decreasing epidemiologic load*.

Corresponds to the period after the peak of the *Pandemic* phase and up to its end (WHO) or to *Deceleration* stage (CDC) (Figure 1). Less stringent restriction measures (lower stringency index) are imposed.

Recommendation: Spirometry and other low-risk PFTs (Table 1) in selected cases (Table 3) can be performed.

- *Low epidemiologic load*.

**Table 3:** Patient selection criteria for performing PFTs during the COVID-19 pandemic.

**Epidemiologic load**	**Patient selection**
Moderate and increasing or high	None (suspension of all PFTs)
Moderate and decreasing	Stringent criteria
	Only patients for whom urgent management decisions must be taken
Low	Less stringent criteria
	Patients with CF,^*^ poorly controlled asthma, initial asthma diagnosis, asthma that requires treatment modification, patients with chronic lung diseases who require therapeutic interventions, patients with interstitial or restrictive lung disorders, pre-operative evaluation, enrolment on transplant lists
None	All patients (usual criteria)

* *Patients with CF or immunocompromised patients are examined in separate rooms or before any other patients (i.e., at the beginning of the session for the respective day)*.

*PFTs, pulmonary function tests; CF, cystic fibrosis*.

Corresponds to *Transition* (WHO) or to *Preparation* stage (CDC) (Figure 1). Loose restriction measures are imposed.

Recommendation: All PFTs can be performed with less stringent patient selection criteria (Table 3).

- *No epidemiologic load*.

Corresponds to post pandemic phase (Figure 1). No restriction measures are imposed.

Recommendation: All PFTs can be performed with usual selection criteria.

### Universal Clinical Screening Prior to Performing PFTs

Body temperature should be measured in all children and their accompanying family members prior to entering the pulmonary function laboratory, ideally with a non-contact thermometer. Each PFTs candidate should complete a structured history questionnaire, focusing on recent history, COVID-19 related symptoms and suspicious contacts (Supplementary Table 1). Screening with rapid SARS-CoV-2 antigen detection tests (child and accompanying caregiver) may also be considered, depending on local policies. In children with fever, suspicious history, or relevant symptoms, PFTs should be postponed for at least 14 days. After this period, the patient should be re-evaluated (clinical screening); SARS-CoV-2 molecular testing may also be considered. Of note, a negative rapid antigen detection test cannot conclusively rule out the infection (35). High-risk individuals (Supplementary Table 1) should not undergo PFTs, irrespective of the rapid antigen detection test results.

## Special Measures of Protection

### Appointment

All tests should be scheduled according to the instructions of the regional/national health authorities.

Specific recommendations:

- An interval of at least 30 min should be maintained between consecutive appointments.
- When possible, clinical pre-screening should be applied via telephone or email. PFTs should not be scheduled in children reporting fever, suspicious contacts, or relevant symptoms.
- In case an urgent test is required (e.g., sudden loss of asthma control), the laboratory timetable should be rearranged so that the time interval of 30 min between consecutive patients is guaranteed.
- In special cases (e.g., patients with CF, immunocompromised patients, or patients with severe chronic lung disease), the children should be ideally examined in separate laboratory rooms or before any other patients (e.g., at the beginning of the session for the specific day).

### Patient Reception

The instructions of national/regional health authorities regarding patient's reception and waiting in dedicated areas (i.e., use of face masks, hand hygiene, social distancing, etc) should be strictly abided by.

Specific recommendations:

- In the waiting room/area should remain one patient with one accompanying person.
- Structured clinical screening (Supplementary Table 1) should be carried out for every patient upon arrival.
- All patients and accompanying persons should use appropriate hand disinfectant upon entering the laboratory.
- All patients and accompanying persons should use face masks (surgical or similar) during their stay in the laboratory.
- Patients and the accompanying persons should be advised to avoid touching any surfaces or objects in the laboratory.

### Laboratory Space Arrangements

- Ideally, pulmonary function testing should be performed in dedicated, separate rooms.
- If this is not possible, it is advised to isolate the space where PFTs are performed, for e.g., by plexiglass or similar partition panels.
- In addition:
  - PFTs should be performed near a well-ventilated spot, for example an open window.
  - The exhaled air should be directed toward a neutral surface (e.g., a wall or a partition panel) that can be easily disinfected upon test completion. Alternatively, the patient should be facing an open window during the procedure.
- Bronchoprovocation tests and cardiopulmonary exercise tests should only be performed in dedicated, separate rooms, provided that the epidemiologic load is low.

### Personal Protective Measures

The instructions of national/regional health authorities regarding personal protective equipment (PPE) should be strictly abided by.

Specific recommendations:

- The minimum necessary personnel should be present in the laboratory.
- All staff members should use PPE as advised by regional/national health authorities (e.g., face masks, disposable gloves, etc).
- Particularly the examiner should use:
  - Face shield or face goggles.
  - Face mask, at least of type FFP2 (Europe), KN95 respirator (Asia), or KN95 respirator (USA).
  - Disposable gloves; meticulous hand washing is advised before and after use.
  - Protective disposable gown (plastic or similar).

The examiner should avoid contact with laboratory surfaces and objects while wearing PPE.

After test completion, non-reusable equipment should be disposed in special bins.

### Performing the Test

Pulmonary function tests should be carried out according to the guidelines (36, 37).

Specific recommendations:

- The procedure is thoroughly explained to the patient, preferably by using video resources or leaflets; live demonstration by the examiner is discouraged.
- The patient should remove the face mask immediately prior to the test and put it in place immediately after the completion of the maneuvers. Should multiple attempts be required, the mask must be worn between these attempts.
- The test should always be carried out using disposable, in-line filters, with incorporated mouthpiece (i.e., mouthpiece as integral part of the filter) (3, 36– 38). Ideally, the shape of the mouthpiece should prevent air loss during forced respiratory maneuvers. A bacterial/viral filtration capacity of at least 99.8% is advisable; most commercially available filters meet this specification. The filter should be discarded in a special bin following test completion. Some laboratories use reusable mouthpieces and/or filters with reusable housing but disposable pads. All reusable parts should be meticulously cleaned and disinfected between patients; if complete disinfection is not possible, these parts should not be reused on the same day (3, 38).
- Use of nasal clips is necessary. Single use, disposable clips are preferred. Should non-disposable clips be chosen, they can be reused following meticulous cleaning and disinfection between patients.
- It is advised that the examiner stands behind the patient to avoid any contact with the stream of blown air.
- If reversibility testing is deemed necessary, it should only be carried out using pressurized metered dose inhalers and the patient's personal holding chamber. If the laboratory's holding chamber is used, it should be subsequently cleaned and disinfected according to the instructions of the manufacturer, and not be reused on the same day. Use of nebulizers is not advisable.

### Cleaning and Disinfection

- Testing equipment and laboratory surfaces should be meticulously cleaned between appointments.
- Equipment cleaning and disinfection should strictly adhere to manufacturer's instructions (type of disinfectants, procedure, etc). Disinfection of exposed electronic parts (e.g., sensors) or difficult to approach circuits merits special attention. Should the manufacturer advise against frequent cleaning or the procedure is difficult and time consuming, pulmonary function testing should be performed judiciously or even suspended.
- Generally, solutions containing 65–75% ethanol, >0.1% sodium hypochlorite or >0.5% hydrogen peroxide destroy the virus within a few minutes. The application of sodium hypochlorite requires attention since its vapors are particularly irritative and may harm the lungs (39). It is advisable to use the solution in adequately ventilated rooms and only when the patients are not present.
- All proximal surfaces must be cleaned immediately after the test. Special attention should be given to surfaces that are not directly related to the test but can easily be cross-infected (e.g., computer keyboards).
- Natural room ventilation (i.e., keeping windows open) is recommended during the test and for the following 15 min.
- Use of air-conditioning is discouraged unless the space is ventilated by a central air-conditioning unit with separate inward and outward air ducts.

## Conclusion

Following an extensive review of the relevant literature, we present the HPRS recommendations on pulmonary function testing in children and adolescents during the COVID-19 era and beyond. We recommend that PFTs should be carried out after careful evaluation of the epidemiologic load, structured clinical screening of all candidates, and application of special protective measures to minimize the risk of viral spread and cross infection. These principles have been integrated into a dynamic action plan that may adapt to local epidemiologic circumstances at the local, regional, or national level, thus permitting PFTs performance during the pandemic safely and affectively. Further research on the risk of SARS-CoV-2 spread in the pulmonary function laboratory is required to create evidence-based guidelines for protecting both patients and health care personnel performing PFTs.

## Author Contributions

SF: conceptualization (lead) and writing—original draft (equal). DG: writing—original draft (lead) and resources (supporting). NK, HO, EA, KD, FK, EP, MA, and MM: writing—review and editing (equal) and resources (supporting). IT: writing—review and editing (lead) and (supporting). All authors approved the final manuscript as submitted and agree to be accountable for all aspects of the work.

## Members of the Hellenic Pediatric Respiratory Society

Alexopoulos Emmanouil, Anagnostopoulou Penelopi, Anthracopoulos Michael, Charisi Martha, Chatsiparasidis Grigorios, Chatzimichael Athanasios, Chrisochoou Elissabet-Anna, Dermitzaki Evi, Dimitriou Gabriel, Douros Konstantinos, Eboriadou-Petikopoulou Maria, Fouzas Sotirios, Galogavrou Maria, Gidaris Dimos, Gogou Maria, Grammeniatis Valisios, Haidopoulou Aikaterini, Hatziagorou Elpis, Kaditis Athanasios, Kalampouka Efthymia, Kampouras Asterios, Karampatakis Nikolaos, Karantaglis Nikolaos, Kasimos Dimitrios, Katsara Maria, Katsardis Charis, Kavvadia Valia, Kirvassilis Fotios, Koltsida Georgia, Kontouli Kalliopi, Koumpourlis Anastasios, Lampropoulos Panagiotis, Lariou Maria-Stella, Loukou Ioanna, Makariou Ioannis, Matzourani Evaggelia, Nousia Lemonia, Opsimos Charalampos, Panagiotopoulou-Gartagani Polytimi, Papadopoulos Marios, Papadopoulou Athina, Paraskakis Emmanouil, Polychronakis Theofilos, Priftis Kostas, Sakelaropoulou Afroditi, Tanou Kalliopi, Triantou Aikaterini, Tsampouri Sofia, Tsanakas Ioannis, Tsartsali Lemonia, Tsiligiannis Theofanis, Valeri Rozalia, Vervenioti Aggeliki, Yialouros Panayiotis.

## Conflict of Interest

The authors declare that the research was conducted in the absence of any commercial or financial relationships that could be construed as a potential conflict of interest.
