# Supplementary material for: Pediatric Pulmonary Function Testing in COVID-19 Pandemic and Beyond. A Position Statement From the Hellenic Pediatric Respiratory Society
Source: Front Pediatr. 2021 May 21;9:673322. doi: 10.3389/fped.2021.673322 (PMC8175853; doi:10.3389/fped.2021.673322)
Supplement: Supplementary file 1 [file Table_1.DOCX]

| **Supplementary Table 1.** Structured clinical screening prior to pulmonary function testing during the COVID-19 pandemic | | | | | | | |
| --- | --- | --- | --- | --- | --- | --- | --- |
| **Given & Family Name:** | | | | | | **Date:** | |
| **Diagnosis:** | |  | | | | | |
| **Temperature (child):** | | | **^o^C** | **Temperature (caregiver):** | | | **^o^C** |
|  | | | | | | | |
| **Has the patient had any of the following during the last 2 weeks?** | | | | | | | |
|  | Temperature >37.5 ^o^C | | | | Comments: | | |
|  | Nasal congestion | | | |  | | |
|  | Sore throat | | | |  | | |
|  | Conjunctivitis | | | |  | | |
|  | Cough | | | |  | | |
|  | Myalgias / arthralgias | | | |  | | |
|  | Vomiting – diarrhoea | | | |  | | |
|  | Taste or smell disorders | | | |  | | |
|  | Contact with a COVID-19 confirmed or suspected case | | | |  | | |
|  | Other symptoms | | | |  | | |
| **Has any family member had any of the above symptoms during the last 2 weeks?**  □ Yes □ No | | | | | | | |
|  | | | | | | | |
| **Has any family member had contact with a COVID-19 confirmed or suspected case during the last 2 weeks?**  □ Yes □ No | | | | | | | |
